# Neighborhood Socioeconomic Status, Depression, and Health Status in the Look AHEAD (Action for Health in Diabetes) Study

**DOI:** 10.1186/1471-2458-11-349

**Published:** 2011-05-19

**Authors:** Tiffany L Gary-Webb, Kesha Baptiste-Roberts, Luu Pham, Jacqueline Wesche-Thobaben, Jennifer Patricio, F Xavier Pi-Sunyer, Arleen F Brown, LaShanda Jones-Corneille, Frederick L Brancati

**Affiliations:** 1 Department of Epidemiology, Columbia Mailman School of Public Health, New York, NY, USA; 2 Department of Epidemiology, Johns Hopkins Bloomberg School of Public Health, Baltimore, MD, USA; 3 Division of General Internal Medicine, Johns Hopkins School of Medicine, Baltimore, MD, USA; 4 Department of Biostatistics, Johns Hopkins Bloomberg School of Public Health, Baltimore, MD, USA; 5 Division of Internal Medicine, University of Pittsburgh, Pittsburgh, PA, USA; 6 Department of Medicine, St. Luke's--Roosevelt Hospital Center, New York, NY, USA; 7 Division of General Internal Medicine and Health Services Research, Department of Medicine, David Geffen School of Medicine at UCLA, Los Angeles, CA, USA; 8 Department of Psychiatry, University of Pennsylvania, Philadelphia, PA, USA

## Abstract

**Background:**

Depression and diminished health status are common in adults with diabetes, but few studies have investigated associations with socio-economic environment. The objective of this manuscript was to evaluate the relationship between neighborhood-level SES and health status and depression.

**Methods:**

Individual-level data on 1010 participants at baseline in Look AHEAD (Action for Health in Diabetes), a trial of long-term weight loss among adults with type 2 diabetes, were linked to neighborhood-level SES (% living below poverty) from the 2000 US Census (tracts). Dependent variables included depression (Beck Inventory), and health status (Medical Outcomes Study (SF-36) scale). Multi-level regression models were used to account simultaneously for individual-level age, sex, race, education, personal yearly income and neighborhood-level SES.

**Results:**

Overall, the % living in poverty in the participants' neighborhoods varied, mean = 11% (range 0-67%). Compared to their counterparts in the lowest tertile of neighborhood poverty (least poverty), those in the highest tertile (most poverty) had significantly lower scores on the role-limitations(physical), role limitations(emotional), physical functioning, social functioning, mental health, and vitality sub-scales of the SF-36 scale. When evaluating SF-36 composite scores, those living in neighborhoods with more poverty had significantly lower scores on the physical health (β-coefficient [β] = -1.90 units, 95% CI: -3.40,-0.039), mental health (β = -2.92 units, -4.31,-1.53) and global health (β = -2.77 units, -4.21,-1.33) composite scores.

**Conclusion:**

In this selected group of weight loss trial participants, lower neighborhood SES was significantly associated with poorer health status. Whether these associations might influence response to the Look AHEAD weight loss intervention requires further investigation.

## Background

Despite the surge of literature evaluating the neighborhood environment and general health, there is only a smaller, growing body of literature evaluating neighborhood and mental health outcomes [1]. Most previous studies have examined the perception of neighborhood problems with depression, anxiety, and health status [2-7], with only a few using objective measures of the neighborhood environment [8]. Overall, most studies were multi-level and showed that more neighborhood problems or detrimental aspects of the physical environment are associated with worse mental health, particularly, depression.

It is well known that individuals with diabetes experience more depression and diminished health status compared to those without diabetes [9,10]. Therefore, to explore potential contributions to this relationship, it is important to understand the association between objective neighborhood context, health status, and depression among individuals with diabetes. We conducted a multi-level, cross-sectional analysis at baseline in the Look AHEAD study (a multicenter controlled trial in 5,145 overweight adults with type 2 diabetes designed to determine the long-term health effects of interventions to achieve intentional weight loss) to determine the association between neighborhood and weight-related health behaviors [11]. Results showed several significant associations with neighborhood and weight control behaviors for food and physical activity. In this manuscript, we evaluated the association between neighborhood poverty and the expanded outcomes of individual-level health status and depression.

## Methods

### Study Population of the Parent Study

The primary objective of the Look AHEAD (Action for Health in Diabetes) study [12,13] is to examine, in overweight volunteers with type 2 diabetes, the long-term effects of an intensive lifestyle intervention program designed to achieve and maintain weight loss by decreased caloric intake and increased physical activity. The intervention group is compared to a control condition involving a program of diabetes education and support. The primary basis for the comparison is the incidence of serious cardiovascular events. Other outcomes, including cardiovascular disease risk factors, diabetes-related metabolic factors and complications, and the cost-effectiveness of the intensive intervention are also studied. Participants are 5,145 volunteers with type 2 diabetes who are 45-75 years of age and overweight or obese (body mass index [BMI] ≥ 25 kg/m^2^).

### Study Population of the Ancillary Study

This ancillary study was conducted using baseline (before the intervention) data from Look AHEAD participants at 4 clinical sites; Baltimore(n = 302), Philadelphia(n = 293), Pittsburgh(n = 321), and New York(n = 303). Sites were chosen because of their close geographic proximity relative to all of the Look AHEAD clinical sites and similar demographic profile (predominately white and African-American). The total study sample for this ancillary consists of 1010 participants with complete data on neighborhood environment and other key variables. Addresses were used to identify the corresponding census tracts for each participant (neighborhood) as defined by the 2000 US Census using a process called geocoding and software program ArcGIS™. The program matches imported addresses to geographic maps and other geographic data. Matches are rated with scores from 0 (no match) to 100 (perfect match); we accepted matches with 80% certainty or more. Once we identified the census tracts and corresponding data for each participant, these data were linked to the individual-level participant data collected during the Look AHEAD trial.

### Main Data Sources

Data are derived from the 2000 US Census long form and include demographic characteristics (age, race, sex), housing characteristics (housing structure, number of rooms), economic characteristics (occupation, place of work and journey to work) and financial characteristics (value of home, rent, utilities cost) for each census tract.

Participants in the Look AHEAD study underwent extensive data collection at baseline, including interview, physical examination, and blood and urine assays [12]. Although the trial will last over 10 years, this manuscript is restricted to data collected at baseline only. The Look AHEAD trial was approved by the Johns Hopkins School of Medicine Institutional Review Board.

### Key Independent Variables

Using the census data, indices of neighborhood socio-economic status (SES) developed by Diez-Roux and Winkleby/Cubbin were created using variables such as the % of persons living below poverty, % of adults with a college degree, median household income, % of persons earning interest income, % of adults in executive/managerial occupations, and % of adults who are unemployed. To produce comparable data for the Diez-Roux and Cubbin indices used in previous studies [14-16], we presented them along with the single item "% of individuals in the census tract living below the federal poverty line" because this measure is highly correlated with other census-based indices and has been shown to be similarly predictive of health outcomes [16].

Covariates were individual-level socio-demographic characteristics: sex, age in years, education in years, race (black, White, Hispanic or other), and categories of yearly, personal income. Body Mass Index (BMI) was presented to show that, per Look AHEAD eligibility criteria, all participants were overweight or obese.

### Key Dependent Variables

The Medical Outcomes Study (MOS) Short Form-36 Health Survey (SF-36), a multidimensional scale of health status designed for self or interviewer administration was used to measure health status [17,18]. The SF-36 has demonstrated reliability and validity and is widely used in health outcomes research [19]. The SF-36 measures 8 health domains: 1) Physical functioning, 2) Role limitations because of physical health problems, 3) Bodily pain, 4) Social functioning, 5) General mental health (psychological distress and psychological well-being, 6) Role limitations because of emotional problems, 7) Vitality (energy/fatigue), and 8) General health perceptions. SF-36 responses were recorded on 5-point scales. Scores for each health domain scale range from 0 to 100, with higher scores indicating better functioning or well being. Composite scores for global, physical, and mental health were also calculated [19]. Depressive symptoms were measured using the Beck Depression Inventory [20]; higher scores indicate more depressive symptoms.

### Statistical Analysis

In this analysis, the main independent variables were the neighborhood factors and the main dependent variables were individual-level depression and health status from the Look AHEAD study. Descriptive statistics were used to describe the study population.

Multi-level linear models were used to analyze the aggregate and individual level data [21-23]. Recognizing that when studying group-level variables, individuals are nested within those groups, multi-level analyses are designed to account for this clustering. In the current study, intercept terms were allowed to vary for each cluster (random effect) while all other variables were considered as fixed effects. Multilevel models were used specifying census tract as the cluster variable. They were fit first with the neighborhood level factor (% poverty) as the independent variable and individual-level depression and health status as outcome variables in separate models. Subsequently, individual-level SES (personal yearly income and education) were added while also controlling for potential confounders (age, sex, race). This enabled us to determine the independent contribution of neighborhood SES entered into the models as tertiles and β coefficients comparing the highest tertiles (most poverty) to the lowest tertile (least poverty) are shown in the table.

We also examined if neighborhood was associated with weight and a number of other clinical variables (including glycemic control). Since this ancillary study was set within a randomized controlled trial at baseline, the participants had similar health profiles at the beginning of the study and most of those associations were not significant. Therefore, we did not feel that it was appropriate to adjust for these variables in the analysis. All analyses were conducted using STATA statistical software, version 10.

## Results

### Selected Baseline Characteristics of Study Participants

Selected baseline characteristics of the study participants are presented in Table 1. Participants were on average 59.2 ± 6.7 years of age and 42% male. The majority were white (64.4%), 27% were Black/African American, and 8.3% were of "other" races. About a third of participants had at least some college education and about 52% had a college education or more; the majority of participants had annual income >$40,000. All participants were at least overweight or obese (BMI >25 kg/m^2^), eligibility criteria for Look AHEAD. Participant neighborhoods were diverse. Of all the neighborhoods represented in the study, the mean % of those living below the federal poverty level was 11%. Overall, there were 920 unique census tracts represented in the study; Baltimore = 201, New York = 257, Philadelphia = 245, Pittsburgh = 217. The number of participants per census tract ranged from 1-6.

**Table 1:** Selected Characteristics of 1010 Look AHEAD Participants

Socio-demographic Characteristics	
Age (years)	59.2 ± 6.7
Sex	
Male	421 (41.7)
Education (years)	
≤12	161 (15.9)
13 - 16	329 (32.6)
16+	520 (51.5)
Race	
Black	276 (27.3)
White	650 (64.4)
Other or Hispanic	84 (8.3)
Income	
<$20,000	74 (7.3)
$20,000-$40,000	181 (17.9)
$40,000-$60,000	211 (20.9)
$60,000-$80,000	179 (17.7)
≥$80,000	365 (36.2)
Body mass index [BMI, kg/m^2^]	36.2 ± 5.8
Overweight [25-29.9]	132 (13.1)
Obese [30-39.9]	640(63.4)
Extreme Obesity[≥40]	238 (23.5)
**Neighborhood Census Tract Indicators**	
Percent Below Poverty [Range = 0, 0.67]	0.11 ± 0.10
Tertile 1 (<0.05)	
Tertile 2 (0.05-0.11)	
Tertile 3 (>0.11)	
Cubbin Deprivation Score [Range = -2.9, 2.8]	-0.006 ± 0.79
Tertile 1 (<-0.34)	
Tertile 2 (-0.34-0.31)	
Tertile 3 (>0.31)	
Diez-Roux Deprivation Score [Range = -18.7, 12.0]	0.003 ± 5.1
Tertile 1 (<-2.00)	
Tertile 2 (-2.00-2.59)	
Tertile 3 (>2.59)	
**Depression**	
Beck Depression Score [Range = 0, 30] *	4.72 ± 4.39
**Health Status**	
Physical Functioning [Range = 19, 58]	48.51 ± 7.85
Role Limitations-Physical [Range = 26, 56]	44.03 ± 12.07
Bodily Pain [Range = 22, 60]	49.59 ± 8.68
General Mental Health [Range = 23, 60]	49.40 ± 6.78
Role Limitations-Emotional [Range = 19, 54]	46.58 ± 12.37
Social Functioning [Range = 17, 57]	52.77 ± 7.60
Vitality [Range = 29, 65]	49.98 ± 6.81
General Health [Range = 23, 64]	45.89 ± 8.37
General Health Composite Score [Range = 22, 61]	45.63 ± 8.89
Mental Health Composite Score [Range = 23, 62]	50.08 ± 7.53
Physical Health Composite Score [Range = 20, 63]	47.77 ± 8.17

*N = 948

All results presented as N(%) or mean ± SD

Higher Cubbin and Diez-Roux score = lower SES

### Association between Neighborhood SES, Health Status, and Depression

Those participants living in neighborhoods with more poverty (highest tertile) had significantly lower scores on the role limitations-physical, role limitations-emotional, physical functioning, social functioning, mental health and vitality sub-scales of the SF-36 health status measure (data not shown). When evaluating SF-36 composite scores (Table 2), those living in neighborhoods with more poverty had significantly lower scores on the physical health (β-coefficient [β] = -1.90 units, 95% CI: -3.40,-0.039), mental health (β = -2.92 units, -4.31,-1.53) and global health (β = -2.77 units, -4.21,-1.33) composite scores. Likewise, those in neighborhoods with more poverty had higher scores (indicating worse symptoms) on the Beck Depression Inventory (β = 0.68 units, -0.12, 1.48), although this finding was not statistically significant. No significant differences were shown for those in the middle tertile of poverty compared to the lowest for health status or depression. Although the associations between the Cubbin and Diez-Roux neighborhood scores and health status were in the hypothesized direction, few were statistically significant.

**Table 2:** Adjusted Beta Coefficients and 95% Confidence Intervals for Neighborhood Indicators, Composite Health Status and Depression among Participants in the Look AHEAD Study

		Physical Health Composite	Mental Health Composite	Global Health Composite	Beck Depression
Below Poverty	2	0.42	-0.48	-0.06	-0.02
(%)		(-0.84, 1.69)	(-1.49, 0.54)	(-1.18, 1.06)	(-0.67, 0.63)
	3	**-1.90**	**-2.92**	**-2.77**	0.68
		**(-3.40, -0.39)**	**(-4.31, -1.53)**	**(-4.21, -1.33)**	(-0.12, 1.48)
Cubbin Score	2	-0.66	0.06	-0.30	**-0.68**
		(-1.97, 0.65)	(-1.07, 1.19)	(-1.52, 0.92)	**(-1.37, 0.00)**
	3	-0.47	-0.78	-0.78	-0.58
		(-1.98, 1.04)	(-2.11, 0.55)	(-2.18, 0.62)	(-1.36, 0.19)
Diez-Roux Score	2	-0.27	0.13	-0.04	**-0.71**
		(-1.58, 1.04)	(-1.00, 1.26)	(-1.26, 1.17)	**(-1.40, -0.02)**
	3	-0.56	-0.85	-0.85	-0.52
		(-2.15, 1.02)	(-2.25, 0.55)	(-2.34, 0.64)	(-1.31, 0.27)

All models adjusted for age, sex, education, personal yearly income and race

^†^All represent comparisons to tertile 1 (reference groups)

Higher Cubbin and Diez-Roux score = lower SES

## Discussion

Our results suggest that among this group of overweight adults with type 2 diabetes in the Look AHEAD study, lower neighborhood SES was significantly associated with poorer health status. These conclusions are supported by results from this study that included a diverse range of neighborhoods, detailed individual-level data, and a large percentage of minority participants.

There were, however, a few limitations. First, using the census tract as a proxy for neighborhood has been criticized, however, many studies have used this indicator, allowing us to compare our findings across studies. Furthermore, the wealth of data available from the US Census provides a comprehensive view of this geographic entity. Similarly, the neighborhood data may not have represented the entire baseline time-period for the Look AHEAD study. Data used were from the 2000 Census and 2004 Consumer database; Look AHEAD participants were recruited from 2001-2004. Neighborhoods are constantly changing, however the time-frame for the data used was close to the study recruitment period. Second, given the eligibility criteria for entry into the study, the population was fairly homogeneous with respect to some factors. One example was weight and clinical variables such as glycemic control, which had little variation by neighborhood. In a future study, we plan to conduct longitudinal analyses and determine how neighborhood influences response to the weight loss intervention. The longitudinal analyses should show more variation in the dependent variables as individuals respond differently to the intervention.

## Conclusion

This study supports the previous literature and gives more evidence for a consistent association between neighborhood poverty and its association with poorer mental health outcomes. Moreover, it gives weight to a strong association when using an objective measure of neighborhood SES which supports the prior studies that focused mostly on perceived measures of neighborhood. Furthermore, this study was conducted in a sample of adults with type 2 diabetes. Persons with diabetes are known to have higher rates of depression [9,10,24], and understanding how other factors influence depression in this population will ultimately contribute to strategies for prevention.

Two recent systematic review articles summarized the state of the literature on neighborhood and mental health, particularly in relation to depression or depressive symptoms [25,26]. Although the consistent theme was that few studies attempted to quantify potential mechanisms, many different pathways and mediating variables were hypothesized. For example, Kim and colleagues outlined a conceptual framework that considered several pathways as potential mediators including: 1) physical health as a result of environmental hazards, 2) health behaviors that may be inhibited by features of the physical environment, 3) psychosocial stress as a result of neighborhood disorder and crime, and 4) resources and social capital that might be lacking as a result of suboptimal social environment [26,27]. Mair and colleagues support these hypotheses and suggest that developing more theory on these process features and empirically testing them is fundamental to strengthening causal inference [25]. Consequently, future studies should pay careful attention to these mechanisms order to identify areas for intervention. A recent qualitative assessment using concept mapping has begun this process [1] and should provide the foundation for further development.

## Competing interests

The authors declare that they have no competing interests.

## Authors' contributions

TLG-W and FLB, were responsible for the conception and design of the study. KB-R was responsible for the data management and data analysis. LP advised on the statistical analysis. TLG-W drafted the manuscript. KB-R, JW-T, JP, FP-S, AFB, LJ, FLB were responsible for critical review of the manuscript and interpretation of findings. All authors are responsible for the final version of the manuscript.

## Pre-publication history

The pre-publication history for this paper can be accessed here:

http://www.biomedcentral.com/1471-2458/11/349/prepub
